# 

**Published:** 1905-07-15

**Authors:** 


### NOTICE TO CORRESPONDENTS.

All MSS., Letters. Books for Review, and other matters intended for the Editor should be addressed The Editor, "The Hospital," The Hospital Buildings, 28 & 29 Southampton Street, Strand, W.C.

The Editor cannot undertake to return rejected MS., even when accompanied by stamped directed envelope.

### Notice to Advertisers and Subscribers.

All Advertisements, Orders for Copies of The Hospital, and Business Communications should be addressed to **THE MANAGER (Not to the Editor),** The Hospital, 28 & 29 Southampton Street, Strand, London, W.C.

The Cost of Subscription, post free, is at follows :—Per week, 3½d.; per uarter, 3s. 3d.; per half-year, 6s. 6d.; per annum, 13s. Foreign and olonial :—Per annum, 19s. 6d., payable, in all cases, in advance.

**NOTICE.—Vol. XXXVII. of "The Hospital" October 1904 to March 1905), in handsome cloth binding, will shortly be on sale at the office of this Journal, price 10s. 6d. Cases for binding the Numbers contained in this Volume 2s. Index to Vol. XXXVII., 3½d., post free.**

**The Monthly Part for July is now ready. Price 1s., by post, 1s. 3d.**

### MEDICAL APPOINTMENTS.

**W. B. Barclay**, L.R.C.P. & S.Edin., Certifying Surgeon under the Factory and Workshop Act for the Aldershot District of the County of Hants. **Frederick E. Batten**, F.R.C.P.Lond., Physician to the Hospital for Sick Children, Great Ormond Street. **Richard Kingston Bird**, L.R.C.P. & S.Edin., L.F.P.S.Glasg., Acting Officer of Health for the shire of Arapiles, Victoria, Australia. **Robert Lee Brown**, L.R.C.P. & S.Edin., L.F.P.S. Glasg., Acting Officer of Health for the shire of Cranbourne, Victoria, Australia. **John Roger Charles**, M.A., M.D., B.C.Cantab., M.R.C.P.Lond., M.R.C.S.Eng., Honorary Assistant Physician to the Bristol Royal Infirmary. **J. F. Dobson**, M.S.Lond., F.R.C.S.Eng., Honorary Assistant Surgeon to the General Infirmary at Leeds. **John Raynor Hatfield**, L.R.C.P. & S.Edin., L.F.P.S.Glasg., Medical Officer and Public Vaccinator for the Kingskerswell District, by the Newton Abbot (Devon) Board of Guardians. **Alfred Stroud Hosford**, M.R.C.S.Eng., L.R.C.P.Lond., Clinical Assistant to the Evelina Hospital for Sick Children, Southwark Bridge Road, London, S.E. **E. W. Jollye**, M.R.C.S., L.S.A., Certifying Surgeon under the Factory and Workshop Act for the Donington District of the County of Lincoln. **John Jones**, L.R.C.P.Edin., L.F.P.S.Glasg., Certifying Surgeon under the Factory and Workshop Act for the Dolgelly District of the County of Merioneth. **Henry Laurie**, M.D.Melb., Honorary Assistant Physician at the Alfred Hospital, Victoria, Australia. **J. C. G. Ledingham**, M.A., B.Sc., M.B.Aberd., Assistant Bacteriologist to the Serum Department, Lister Institute, Elstree. **W. Jackson Hooker Macgilvray**, L.R.C.P. & S.Edin., Medical Officer to the General Friendly Collecting Society for Higher Broughton, Manchester, and District. **L. A. Moore**, L.R.C.P.Lond., M.R.C.S., Dental Anæsthetist at the Bristol Royal Infirmary. **L. N. Morris**, L.R.C.P.Lond., M.R.C.S., Assistant House Surgeon at the Bristol General Hospital. **Frank J. Nicholls**, M.B., B.C.Cantab., M.R.C.S., L.R.C.P.Lond., by the London County Council, Medical Officer for District No. 25, Woolwich Borough. **W. Oliver**, L.R.C.P., L.R.C.S.Edin., Certifying Surgeon under the Factory and Workshop Act for the Coxhoe District of the county of Durham. **Lloyd Davenport Parry**, L.R.C.S.Edin., Government Medical Officer and Vaccinator at Picton, New South Wales, Australia. **E. Percy Paton**, M.D.Lond., Examiner in Anatomy to the Society of Apothecaries of London. **F. C. H. Piggott**, Surgeon to the Teignmouth (Devon) Branch of the Rational Friendly Society. **E. Dunbar Pownroe**, L.R.C.P.Lond. M.R.C.S., Resident Medical Officer at the Winsley Sanatorium. **A. G. Richardson**, M.B., C.M.Edin., Certifying Surgeon under the Factory and Workshop Act for the Rhayader District of the county of Radnor. **A. W. Cecil Richardson**, M.B.Lond., Casualty House Surgeon at the Bristol General Hospital. **G. E. Richmond**, M.D., B.S., B.Sc.Lond., D.P.H.Cantab., by the London County Council, Resident Medical Officer to the Rotherhithe Tunnel Works. **William Robinson**, M.D., M.S., F.R.C.S., Honorary Surgeon to the Sunderland Infirmary. **John Stuart Rose**, M.B., Ch.B.Aberd., D.P.H., Assistant Medical Officer of Health, Penang. **F. H. Rudge**, L.R.C.P.Lond., M.R.C.S., Senior House Surgeon at the Bristol Royal Infirmary. **T. Graham Scott**, L.R.C.P.Lond., Third Anæsthetist to the Hospital for Sick Children, Great Ormond Street. **A. L. Sheppard**, M.B., Casualty Officer at the Bristol Royal Infirmary. **Arthur Rendle Short**, M.D., B.S., B.Sc.Lond., House Physician to the Bristol General Hospital. **W. S. Vernon Stock**, M.B., B.S.Lond., L.R.C.P.Lond., M.R.C.S., Honorary Assistant Anæsthetist to the Bristol Royal Infirmary. **J. O. Symes**, M.D.Lond., Physician to Clifton College, Bristol. **A. C. A. Van Buren**, M.D., B.S., L.R.C.P.Lond., M.R.C.S., Senior House Surgeon at the Bristol General Hospital.

### Books for Private and District Nurses.

**A HANDBOOK FOR NURSES.**
By Dr. J. K. Watson.
5/- net. 5/4 post free.

**THE ART OF MASSAGE.**
By A. Creighton Hale.
6/- post free.

**ON PREPARATION FOR OPERATION IN PRIVATE HOUSES.**
By Stanmore Bishop, F.R.C.S.
6d. net. 7d. post free.

**A CASE BOOK FOR PRIVATE NURSING FOR DAY AND NIGHT NURSE.**
48 pages. Size of Exercise Book.
3d. net. 4½d. post free.

**PRACTICAL HINTS ON DISTRICT NURSING.**
By Miss Amy Hughes.
1/- post free.

**NURSING NOTES ON MIDWIFERY AND GYNÆCOLOGY.**
By Sister Ross.
2/- net. 2/1 post free.

**THE "SIMPLEX" DISTRICT NURSES' CASE BOOK.**
For Fifty Cases.
6d. net. 7d. post free.

**THE ART OF FEEDING THE INVALID.**
1/6 post free.

**OUTLINES OF ROUTINE IN DISTRICT NURSING.**
By M. Loane.
New edition, now ready. 1/6 net. 1/9 post free.

**PRACTICAL GUIDE TO SURGICAL BANDAGING AND DRESSINGS.**
By W. Johnson Smith, F.R.C.S.
2/- post free.

**HOME NURSING OF SICK CHILDREN.**

By M. L. Moberly.

1/- post free.

**THE EXAMINATION OF THE URINE.**
By Dr. J. K. Watson.
1/- net. 1/1 post free.

**MODERN NURSING IN PRIVATE PRACTICE.**
By Sir William Bennett, F.R.C.S.
6d. net. 7d. post free.

**POCKET-BOOK OF TEMPERATURE CHARTS.**
17 twelve-hour and 8 four-hour charts, perforated for removal.
6d. net. 7d. post free.

**THE NURSES' PRONOUNCING DICTIONARY OF MEDICAL TERMS AND NURSING TREATMENT.**
2/- post free.

**HOW TO BECOME A CERTIFIED MIDWIFE.**
By E. Appel, M.B.
1/- net. 1/1 post free.

**VISIT BOOKS, TIME BOOKS, LOAN BOOKS FOR DISTRICT NURSES.**

**TEMPERATURE CHARTS, MORNING AND EVENING AND 4-HOUR CHARTS.**
Prices :— (Post or Carriage Free.)

| | | | |
|---|---|---|---|
| 1,000 ... ... | 23/- | 100 ... ... | 2/9 |
| 500 ... ... | 12/- | 50 ... ... | 1/6 |
| 250 ... ... | 6/6 | 25 ... ... | 1/- |

**JAPANNED TIN WASHABLE CHARTHOLDERS.**
1/- each. 9/6 per dozen.

**The Scientific Press, Ltd.**
Publishers and Booksellers of every kind of Nursing Text-Books, Case-Books, and Charts.
28 & 29 SOUTHAMPTON STREET, STRAND, LONDON, W.C.
***SEND FOR NEW CATALOGUE.***

### The NURSING MIRROR.

**The Nurse's Own Paper.**

**Price ONE PENNY Weekly.**

Of all Booksellers and Newsagents, or posted direct from the Office.

*Write for Subscription Rates to The Manager,*

THE NURSING MIRROR, The Hospital Building, 28 & 29 Southampton Street, Strand, London, W.C.

